# Long noncoding RNA MYOSLID promotes invasion and metastasis by modulating the partial epithelial-mesenchymal transition program in head and neck squamous cell carcinoma

**DOI:** 10.1186/s13046-019-1254-4

**Published:** 2019-06-25

**Authors:** Hong-Gang Xiong, Hao Li, Yao Xiao, Qi-Chao Yang, Lei-Lei Yang, Lei Chen, Lin-Lin Bu, Wen-Feng Zhang, Jia-Li Zhang, Zhi-Jun Sun

**Affiliations:** 1 The State Key Laboratory Breeding Base of Basic Science of Stomatology (Hubei-MOST) & Key Laboratory of Oral Biomedicine Ministry of Education, School & Hospital of Stomatology, Wuhan University, Wuhan, China; 2 Department of Oral Maxillofacial-Head Neck Oncology, School and Hospital of Stomatology, Wuhan University, Wuhan, China; 3 Department of Oral Pathology, School and Hospital of Stomatology, Wuhan University, Wuhan, China

**Keywords:** Long non-coding RNA, Partial epithelial to mesenchymal transition, MYOSLID, Prognostic biomarkers

## Abstract

**Background:**

Partial epithelial mesenchymal transition (p-EMT) was found to play a potential role in the initial stage of metastasis in human head and neck squamous cell carcinoma (HNSCC). Some long noncoding RNAs (lncRNAs) have been reported to function as promoters or inhibitors of cancer metastasis. This study aimed to identify p-EMT-related lncRNAs in HNSCC.

**Methods:**

Differentially expressed lncRNAs (DE-lncRNAs) and mRNAs (DEGs) in HNSCC obtained from The Cancer Genome Atlas (TCGA) were screened out by using the “edgeR” package. DE-lncRNAs in the Oral squamous cell carcinoma (OSCC) lncRNA microarray dataset GSE84805 were screened out by using the “limma” package. Slug-related lncRNAs were determined by Pearson correlation analysis (|Pearson correlation coefficient| ≥ 0.4, *p* < 0.01) based on TCGA. Survival analysis were performed for the overlapping DE-lncRNAs by using the “Survival” package. Gene Ontology (GO) and Kyoto Encyclopedia of Genes and Genomes (KEGG) pathway enrichment analyses were used to predict the potential functions of MYOSLID. RT-qPCR and In Site Hybridization (ISH) were used to explore the MYOSLID expression and its clinical significance in HNSCC specimens. Immunohistochemical staining, siRNA, wound healing assay, transwell assay, and western blot were used to explore the biological function and potential molecular mechanisms.

**Results:**

MYOSLID was identified as a Slug-related lncRNA and with prognostic value among the 9 overlapping DE-lncRNAs. GO and KEGG analyses revealed that MYOSLID was closely related to important biological processes and pathways that regulate cancer metastasis. The results of univariate and multivariate Cox regression analysis based on TCGA and HNSCC tissue microarray data suggested MYOSLID was an independent prognostic factor. MYOSLID expression in HNSCC was closely correlated with Slug, PDPN and LAMB3. The knockdown of MYOSLID in OSCC cell line significantly inhibited cell migration and invasion compared to those in the control cells. In addition, the knockdown of MYOSLID significantly reduced Slug, PDPN and LAMB3 expression levels. However, the knockdown of MYOSLID had no effect on the expression levels of the EMT biomarkers E-cadherin and Vimentin.

**Conclusions:**

Our study revealed that MYOSLID expression was closely related to the p-EMT program in HNSCC, and it might be a new predictive biomarker for aggressive HNSCC.

**Electronic supplementary material:**

The online version of this article (10.1186/s13046-019-1254-4) contains supplementary material, which is available to authorized users.

## Background

Head and neck squamous cell carcinoma (HNSCC) is one of the most common human malignancies worldwide that originate from epithelial tissue, more than 90% of head and neck cancer are histopathologically squamous cell carcinoma [1]. An important factor that affects the prognosis of HNSCC is metastasis to the regional lymph nodes or distant organs, which has been reported to reduce the 5-year survival rate by 50% compared with that of patients with early stage disease [2]. Therefore, controlling metastasis is of great importance for fighting cancer. The epithelial to mesenchymal transition (EMT) program has long been considered play an essential role in cancer metastasis [3, 4]. Cancer cells escape from the primary tumor and colonize distant organs to form a second tumor, similar to the process of embryonic cells that travel a long distance and migrate to their destinations and to form an organ [5]. Classical EMT is a complex process in which nonmotile epithelial cells experience a complete loss of apical-basal polarity and cell-cell adhesion properties and transform into mesenchymal cells with the ability to migrate and invade adjacent tissue [3]. The loss of epithelium markers such as E-cadherin and the gain of mesenchymal marker such as Vimentin are the most common hallmarks of EMT [6].

However, scholars have observed that cancer cells at the leading edges of primary tumor in aggressive HNSCC acquire just some of the traits of mesenchymal cells but still retain some of the traits of epithelium cells, which is referred to as partial EMT  (p-EMT) [4, 7, 8]. The implications of a p-EMT program in cancer are still poorly understood. Recent studies show that the most possible implications of p-EMT in squamous cell carcinoma included endowing the leading cells of the cancer nests with the ability to guide a collective migration, which was distinct from a single-cell metastasis, that occurs in a coordinated manner while maintaining cell-cell contact [5]. Podoplanins (PDPN) and laminin subunit beta 3 (LAMB3) were considered as p-EMT markers and have been suggested to be related to cancer cell metastasis. Slug was the only activated epithelial to mesenchymal transition-transcription factor (EMT-TF) in p-EMT cells compared with non-p-EMT cells based on a single-cell sequencing technique [8]. Similarly, Slug was also demonstrated to act as a key regulator of the p-EMT program in a 3D Madin-Darby canine kidney (MDCK) tubule-genesis system that modeled the in vivo process of the p-EMT program [9]. Thus, the precise molecular mechanisms that regulate p-EMT are still not fully understood.

We now understand that lncRNAs driver many important cancer phenotypes by regulating oncogenes or tumor suppressor genes through multiple mechanisms [10, 11]. Many studies have suggested that lncRNAs have the potential to serve as important biomarkers for cancer diagnosis or therapeutic targets for cancer [12]. At present, there are some lncRNAs that have been found to be related to prognosis and metastasis in HNSCC, such as MIR31HG, UCA1, NEAT1, and EGFR-AS1 [13–16]. As EMT is a key and necessary process in the early stage of cancer metastasis, many lncRNAs have been identified with the ability to affect the EMT program by regulating the expression of Slug in different kinds of cancer [17–19]. Studies have show that Slug not only regulate EMT but also play a role in regulating p-EMT [8, 9]. However, lncRNAs that regulate the p-EMT program, along with Slug-related lncRNAs, have never been reported.

We aimed to identify lncRNAs involved in p-EMT in HNSCC. First, we screened differential expressed lncRNAs (DE-lncRNAs) and differential expressed mRNAs (DEGs) in HNSCC from The Cancer Genome Atlas (TCGA) and Gene Expression Omnibus (GEO) databases using the bioinformatics methods. Then we identified lncRNAs that were correlated with the p-EMT regulator gene Slug from the DE-lncRNAs identified from TCGA. We discovered that the lncRNA MYOSLID was the only lncRNA with prognostic value that was correlated with Slug. Next, MYOSLID-related mRNAs was analyzed with Gene Ontology (GO) and KEGG (Kyoto Encyclopedia of Genes and Genomes) analysis, and the results showed that MYOSLID is associated with many important biological functions and signaling pathways that related to metastasis. These results suggested that MYOSLID is a molecule with important functions. Finally, MYOSLID expression and its’ clinical significance in HNSCC was validated with a human HNSCC microarray, and its potential molecular mechanism was explored in oral squamous cell carcinoma (OSCC) cell lines.

## Methods

### TCGA data download and GEO data re-annotation

The RNA sequence data and relevant clinical information of 502 cases of HNSCC and 44 cases of cancer adjacent normal tissues were download from the TCGA database (https://www.cancer.gov/about-nci/organization/ccg/research/structural-genomics/tcga/?redirect=true). The primary site of selected HNSCC cases is larynx, floor of mouth, tonsil, base of tongue, other and unspecified parts of tongue, nasopharynx, gum, oropharynx, hypohparynx, palate, buccal mucosa, lip and other ill-defined sites in oral cavity and mouth.

An OSCC lncRNA microarray profiling dataset (GSE84805) was obtained from the GEO database (https://www.ncbi.nlm.nih.gov/gds/?term=GSE84805). Six paired OSCC cancer tissues and adjacent normal tissues were analyzed in this array (platform: GLP16956, Agilent-045997 Arraystar human lncRNA microarray V3). The GSE84805 lncRNA expression profiling data were acquired by probe reannotation. The steps for reannotation are simply described as follows. First, the probe sequences for the Agilent-045997 Arraystar human lncRNA microarray V3 from the Agilent website (http://www.agilent.com/) were remapped to the human genome (GRCH38) using the SeqMap tool. The probes that were uniquely mapped to the human genome without mismatch were retained. Secondly, we matched the chromosomal location of the retained probes to the chromosomal location of the lncRNAs from the GENCODE project (https://www.gencodegenes.org, release 28).

### HNSCC tissue microarray information and fresh tissue specimen acquisition

The human HNSCC tissue microarray T15–411 included 90 primary HNSCCs, 8 recurrent HNSCCs, 20 HNSCCs with a preoperative chemoradiotherapy history, 37 metastatic lymph nodes, 5 normal mucosa (MUC) samples, and 35 dysplasia (DYS) samples. The clinicopathological information of the patients included in the microarray T15–411 was reported by Wu et al. [20].

In addition, 15 paired fresh OSCC tissues and adjacent normal tissues were obtained from patients with OSCC who underwent surgery at the Department of Oral Maxillofacial Head and Neck Oncology of the Hospital of Stomatology of Wuhan University during 2018.7–2018.12. The clinical information of the 15 OSCC patients is given in Additional file 1: Table S1. All the patients were primary OSCC patients. Residual blood on the fresh tissues was washed away with saline, and the tissue were immediately stored in a − 80 °C refrigerator for total RNA and protein extraction. All patients in this study provided informed consent before surgery. The Medical Ethics Committee of the School and the Hospital of Stomatology of Wuhan University approved the study.

### Identification of DEGs, DE-lncRNAs and the generation of a Venn diagram

Differentially expressed mRNAs and lncRNAs were identified in HNSCC tissues in comparison with normal tissue with data obtained from the TCGA database using the “edgeR” package in R. Differentially expressed lncRNAs were identified in HNSCC tissues in comparison with normal tissues from data obtained from GEO with the accession number GSE84805 using the “limma” package in R. The R scripts of “edgeR” and “limma” were shown in Additional file 2: edgeR.txt and Additional file 3: limma.txt respectively. |Fold Change| > 2 and adjusted *p* < 0.05 were set as the statistical threshold value for differentially expressed mRNAs and lncRNAs. A volcano plot with clustering for the significantly differentially expressed lncRNAs and mRNAs in HNSCC were generated with the “gplots” package in R. A Venn diagram was constructed by using the online website VENNY 2.1.

### Target gene prediction

The coexpression relationships of the lncRNAs and mRNAs were determined by calculating the Pearson correlation coefficients. If the absolute value of the Pearson correlation coefficients between mRNAs and MYOSLID were greater than 0.4, the mRNAs were considered MYOSLID-related mRNAs (*p* < 0.01). Similarly, if the absolute value of the Pearson correlation coefficients between lncRNAs and Slug were greater than 0.4, the lncRNAs were considered as Slug-related lncRNAs (*p* < 0.01).

### Survival analysis

Survival analysis was performed for the overlapping lncRNAs obtained from bioinformatics analysis using the “Survival” package in R. Survival analysis for MYOSLID expression in the HNSCC tissue microarray T15–411 was performed by using the Kaplan–Meier method. The cut-off value of the MYOSLID expression levels was determined based on its median value. The Mantel–Cox log-rank test was used to compare the differences between the two survival curves. *p* < 0.05 was considered to be significant.

### Function enrichment analysis

mRNAs with an absolute value of the Pearson correlation coefficient with MYOSLID that were greater than or equal to 0.3 were included in further function enrichment analysis. The GO and KEGG pathway enrichment analyses were performed with the “R × 64 3.4.1” software using the “clusterProfiler” package. The enriched GO terms and KEGG pathways with a *p* < 0.01 were considered MYOSLID-related biologic processes or signaling pathways.

### RT-qPCR

Total RNA was extracted from tissue samples and cell lines using an RNA prep Pure Tissue Kit and an RNA prep Pure Cell/Bacteria Kit (Tiangen, Beijing, China) according to the manufacturer’s protocols. cDNA was synthesized with a PrimeScript™ RT reagent Kit with gDNA Eraser (Perfect Real Time) (TaKaRa, Dalian, China). Real-time fluorescent quantitative PCR was performed by using the Bio-Rad CFX96 Real-Time PCR Detection System (Bio-Rad, USA). The mixture of PCRs contained TB Green® Premix Ex Taq™ (Tli RNaseH Plus; 12.5 μl), cDNA (2 μl), forward primer (1 μl), reverse primer (1 μl), and RNase-free H_2_O (8.5 μl), with a total volume of 20 μl. The thermal cycling conditions were as follows: 30 s at 95 °C, 5 s for 40 cycles at 95 °C, 30 s at 60 °C. Target mRNA and lncRNA expression was normalized against GAPDH. The differences between groups were calculated with the comparative Ct method (2^−ΔCT^). All experiments were performed in triplicate. Primer sequences are shown in Additional file 1: Table S2.

### Cell culture

The HNSCC cell lines Cal27, SCC4 and SCC9 were purchased from the ATCC (American Type Culture Collection). Tca8113 and the normal human immortalized oral keratinocyte line (HIOEC) from primary normal human oral epithelial cells that were infected with HPV16E6E7 were established at the Ninth People’s Hospital, Shanghai Jiao Tong University School of Medicine [21]. Cal27, SCC4 and SCC9 were cultured in DMEM/high glucose with 10% fetal bovine serum (FBS, Gibco, Grand Island, NY, USA) and 1% streptomycin-penicillin. Tca8113 was cultured in RPMI 1640 (Gibco, USA) supplemented with 10% FBS (Invitrogen, USA) and 1% streptomycin-penicillin. HIOEC cells were cultured in a defined keratinocyte serum-free medium (KSFM; GIBCO BRL, USA).

### In situ hybridization

The expression level of MYOSLID in tissues was measured with digoxigenin-labeled antisense oligonucleotide probes. The slides of the HNSCC tissue microarray were dewaxed and rehydrated. The slides were soaked in citrate solution, heated to boiling for antigen retravel, and incubated with proteinase K (Servicebio, Wuhan, China) at 37 °C for 15 min. Then, the slides were washed 3 times for 15 min each with 0.1 M PBS. The slides were washed with prehybridization buffer for 30 min at room temperature before hybridization with the MYOSLID probe (Servicebio, Wuhan, China, 8 ng/ul) overnight. The sections were then washed with a gradient-diluted SSC solution at 37 °C for 10 min followed by incubation with HRP-labeled mouse anti-digoxigenin (1:1000) (Jackson, USA) for 40 min at 37 °C. Finally, the hybridization signals were visualized with diaminobenzidine (DAB) chromogenic substrate (Dako). The reaction was stopped by washing with water for 5 min. The slide was counterstained with hematoxylin, sealed with neutral resins, and photographed. The probe sequences for MYOSLID were as follows: 5′–DIG- CAGCCATGTCCTTGCCTTCTGCACACGGTA-DIG - 3′.

### Immunohistochemistry and scoring system

Immunohistochemical (IHC) staining of the paraffin-embedded HNSCC tissue microarrays were conducted using primary antibodies against Slug (Cell Signaling Technology, 1:200), LAMB3 (Abcam, 1:250), and Podoplanin/gp36[EPR22182] (Abcam, 1:250), as previously described [22]. An isotype goat IgG antibody was used as a negative control. All slides were scanned with an Aperio ScanScope CS scanner (Vista, CA, USA). The histoscore quantification of each sample was analyzed by the Aperio Quantification software algorithms as previously described [23]. Histoscores of each slide, including ISH stain and IHC stain, were calculated according to the formula (3 + percent cells) × 3 + (2 + percent cells) × 2 + (1 + percent cells) × 1) total intensity/total cell number and were used to assess the histoscore of the pixel quantifications.

### siRNA and transfection

To avoid off-target effects, three siRNAs targeting MYOSLID (GenePharma, Shanghai, China) were synthesized for transfection and to detect the silencing efficiency. Cells were seeded into a 6-well plate (2 × 10^5^ cells/well). siRNAs were mixed with GP-siRNA-Mate Plus (GenePharma, Shanghai, China) at a ratio of 1:1 and incubated for 15 min before being diluted with FBS-free culture medium and then delivered to the HNSCC cell lines. After 48 h of culture, cells were harvested for RNA or protein extraction.

### Matrigel invasion assay

Cell invasion assays were performed with transwell chambers (8.0 μm pore size; Corning, USA), which were coated with 40 μl Matrigel (3× dilution; 40 μl/well; BD Bioscience), in 24-well plates. After transfection for 36 h, the cells were collected and washed with PBS. Then, the cells were resuspended in serum-free medium. A 200 μl cell suspension containing 2.0 × 10^5^ cells were added to the upper chamber, and 600 μl DMEM containing 30% FBS was added to the lower chamber. After 36 h of incubation, the cells on the top of the membranes were removed with a cotton swab, and then the cells underneath the membranes were fixed in 4% paraformaldehyde for 20 min and stained with crystal violet for 30 min. The stained cells that were attached to the membranes were counted under a microscope, and 5 fields of view were randomly selected.

### Wound healing assay

The effect of MYOSLID knockdown on cell migration was evaluated with a wound healing assay. A total of 5 × 10^5^ cells were seeded in 6-well plates containing complete medium and incubated at 37 °C in an atmosphere of 5% CO2 for 24 h. A total of 300 μl of solution containing 5 μl of MYOSLID siRNA and 5 μl of GP-siRNA-Mate Plus was added to each group when the cell density reached 60–70%. A thin scratch was made with a 200 μl pipette tip when the cell density reached 90%, and the complete medium was replaced with a serum-free medium. The width of the scratch was recorded at 0 h, 24 h and 48 h with an inverted microscope (Nikon, Japan) to measure the percentage of the area covered by the migrated cells.

### Western blotting

Cells were lysed in cell lysis buffer (Beyotime Biotechnology, China) containing 1 mM phenylmethylsulfonyl fluoride (PMSF). Total protein concentrations in the supernatant were measured with a bicinchoninic acid assay (BCA) (Beyotime biotechnology, China), and protein (30 μg/lane) was separated on 10% polyacrylamide gels (Servicebio, Wuhan, China) with electrophoresis and subsequently transferred onto PVDF membranes. Membranes were blocked with 5% skim milk in TBST at room temperature for 1 h. The membranes were incubated with primary antibodies against Slug (Cell Signaling Technology, 1:2000), LAMB3 (Abcam, 1:1000), Podoplanin/gp36[EPR22182] (Abcam, 1:2000), E-cadherin (Cell Signaling Technology, 1:1000), and Vimentin (D21H3) XP® (Cell Signaling Technology, 1:1000) overnight at 4 °C. After washing three times for 5 min in TBST, the membranes were incubated for 1 h at 37 °C with an HRP-labeled goat anti-rabbit IgG (Proteintech, Wuhan, China) diluted 1:5000 in TBST. Bands were visualized using a WesternBright Sirius Chemiluminescent Detection Kit (Advansta, California, USA). GAPDH protein levels were used as loading controls. The experiments were repeated three times.

### Statistical analysis

SPSS 19.0 software and GraphPad Prism 8.0 were utilized for statistical analysis. The statistical significance of the differences was determined by a paired *t* test or a Student’s *t* test between two groups. The statistical significance of the differences among three groups was determined by one-way ANOVA. Quantitative data are expressed as the mean ± standard deviation (SD). Pearson’s chi-square test or Fisher’s exact test was used to determine the relationship between MYOSLID expression and the clinicopathological features of the HNSCC patients. Univariate and multivariate analyses were performed to test whether MYOSLID was related to OS. Survival analysis was performed with the Kaplan-Meier and log-rank test. *p* < 0.05 was considered statistically significant.

## Results

### Analysis of the differential expressed lncRNAs with data integrated from the TCGA and GEO databases

**Fig. 1:**
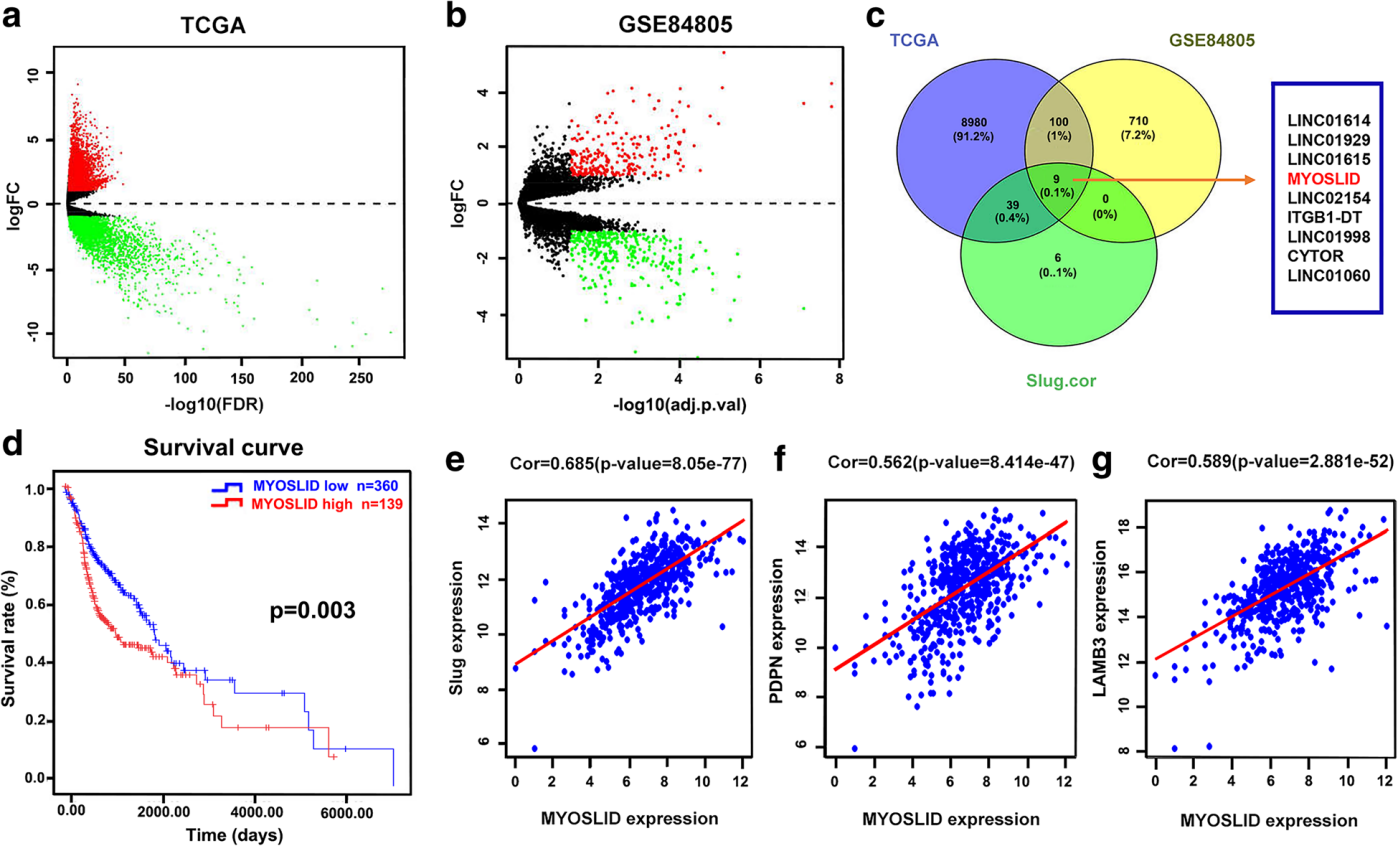
MYOSLID screening and target gene prediction by bioinformatics analysis. **a** Volcano plot of DE-lncRNAs and DEGs in HNSCC from TCGA. **b** Volcano plot of the DE-lncRNAs and DEGs in HNSCC from GEO. **c** Venn diagram of DE-lncRNAs and DEGs common to the TCGA, GEO and Slug-related DE-lncRNAs. **d** Prognosis analysis assay for MYOSLID based on TCGA data. **e**-**g** Target gene prediction was performed by calculating the Pearson correlation coefficient based on TCGA data

A total of 9128 DEGs or DE-lncRNAs, including 5731 upregulated and 3397 downregulated DEGs or DE-lncRNAs, were screened from the TCGA database (|fold change| > 2 and adjusted *p* < 0.05) (Fig. 1a). A total of 819 DEGs or DE-lncRNAs, including 334 upregulated and 485 downregulated (|fold change| > 2 and adjusted *p* < 0.05), were screened from the GEO dataset (Fig. 1b). There were 109 overlapping mRNAs or lncRNAs, including 25 mRNAs and 84 lncRNAs, between the two groups (Fig. 1c).

### Identification of slug-related lncRNAs in human HNSCC based on the TCGA data

lncRNAs with a Pearson correlation coefficient ≥ 0.4 were considered Slug-related lncRNAs. There were 54 lncRNAs identified as Slug-related lncRNAs. There were 9 overlapping lncRNAs between the Slug-related lncRNAs, DE-lncRNAs of the TCGA and DE-lncRNAs of the GEO, which were LINC01614, LINC01929, LINC01615, MYOSLID, LINC02154, ITGB1-DT, LINC01998, CYTOR, and LINC01060 (Fig. 1c). The Pearson correlation coefficient between the MYOSLID and Slug expression levels was 0.685 (*p* = 8.05e-77), which was the maximum correlation coefficient among the 54 Slug-related lncRNAs.

### Survival analysis screening and the assessment of the clinical significance of MYOSLID expression in HNSCC based on the TCGA database

Kaplan-Meier and log-rank tests were performed for the survival analysis of the 9 overlapping lncRNAs. The results showed that only MYOSLID expression was related to survival (log-rank (Mantel-Cox) = 8.809, *p* = 0.003) (Fig. 1d).

Then, we downloaded the clinical information from 502 HNSCC patients from TCGA to further assess the clinical significance of MYOSLID expression. Three HNSCC patients who were lacking most clinical information were excluded from the analysis. The clinical characteristics of 499 HNSCC patients are shown in Additional file 1: Table S3. Then, we analyzed the relationship between MYOSLID and the clinical pathologic parameters using the chi-square test. The results showed that MYOSLID expression was upregulated in HNSCC tissues compared with that in normal tissues (log FC = 2.870, *p* < 0.001). MYOSLID expression was significantly correlated with age (*p* = 0.036), clinical stage (*p* = 0.026) and T classification (*p* = 0.013), as shown in Table 1.

**Table 1:** Relationship between MYOSLID expression and clinicopathological Characteristics of HNSCC patients based on TCGA (*n* = 499)

Characteristics	MYOSLID		χ2	*p* value
	Low expression (*n* = 360)	High expression (*n* = 139)		
Age(y)				
< 60	149	72	4.404	0.036^*^
≥ 60	211	67		
Gender				
Male	257	109	2.534	0.111
Female	103	30		
Race				
White	312	115	1.323	0.724
Black	31	16		
Asian	7	3		
unknow	10	5		
Grade				
G1/2	256	104	1.196	0.754
G3/4	89	31		
GX	13	3		
unknow	2	1		
Stage				
Stage I/II	70	26	7.272	0.026^*^
Stage III/IV	232	103		
unknow	58	10		
T				
T_1_ + T_2_	166	49	8.69	0.013^*^
T_3_ + T_4_	175	87		
unknow	19	3		
N				
Yes	167	69	1.726	0.422
No	173	66		
unknow	20	4		
M				
M0	134	51	1.227	0.747
M1	1	0		
Mx	41	20		
unknow	184	68		
HPV status				
Positive	29	1	9.634	0.002^*^
Negative	49	23		
unknow	282	115		

Note: HNSCC patients were divided into MYOSLID low and MYOSLID high group according to the cut off value (median = 219.8)

^*^p < 0.05 was considered statistic significant. Differences among variables were evaluated by χ2 or Fisher’s exact χ2 -test

The univariate analysis based on the information of the 499 HNSCC patients obtained from TCGA indicated that MYOSLID expression (HR = 1.370, 95% CI [1.012–1.853], *p* = 0.041), gender (HR = 1.381, 95% CI [1.024–1.862], *p* = 0.034), and TNM stage (HR = 1.454, 95% CI [1.118–1.891], *p* = 0.005) were all significantly related to survival. Multivariate Cox regression analysis revealed MYOSLID expression (HR = 1.429, 95% CI [1.045–1.953], *p* = 0.025), gender (HR = 1.372, 95% CI [1.003–1.876], *p* = 0.048), T stage (HR = 1.859, 95% CI [1.224–2.823], *p* = 0.004), N condition (HR = 1.543, 95% CI [1.196–1.990], *p* = 0.001) and HPV status (HR = 1. 374, 95% CI [1.050–1.798], *p* = 0.021) were independent prognostic factors, as shown in Table 2.

**Table 2:** Univariate and multivariate Cox regression analysis for OS in HNSCC patient based on TCGA(*n* = 499)

Variable	Univariate analysis			Multivariate analysis		
	*p*	HR	95% CI	*p*	HR	95% CI
MYOSLID	0.041	1.370	1.012–1.853	0.025	1.429	1.045–1.953
Age	0.088	1.287	0.963–1.720	0.118	1.272	0.941–1.719
Gender	0.034	1.381	1.024–1.862	0.048	1.372	1.003–1.876
Race	0.240	1.218	0.876–1.694	0.808		
Race (1)				0.846	1.155	0.271–4.913
Race (2)				0.892	1.083	0.343–3.419
Race (3)				0.608	1.381	0.403–4.734
Grade	0.872	0.977	0.738–1.293	0.679	0.941	0.705–1.255
Stage	0.127	1.175	0.955–1.445	0.115	0.760	0.541–1.069
T	0.005	1.454	1.118–1.891	0.004	1.859	1.224–2.823
N	0.027	1.300	1.030–1.640	0.001	1.543	1.196–1.990
M	0.039	1.121	1.006–1.249	0.179	1.080	0.965–1.209
HPV	0.024	1.362	1.041–1.782	0.021	1.374	1.050–1.798

Notes: *OS* overall survival, *N* Regional Lymph Nodes, *T* Primary Tumor, *M* distant metastasis

*Abbreviations*: *HR* hazard ratio, *CI* confidence interval

Race = White was defined as reference, Race (1) = Black, Race (2) = Asian, Race (3) = unknow

### GO and KEGG analysis

**Fig. 2:**
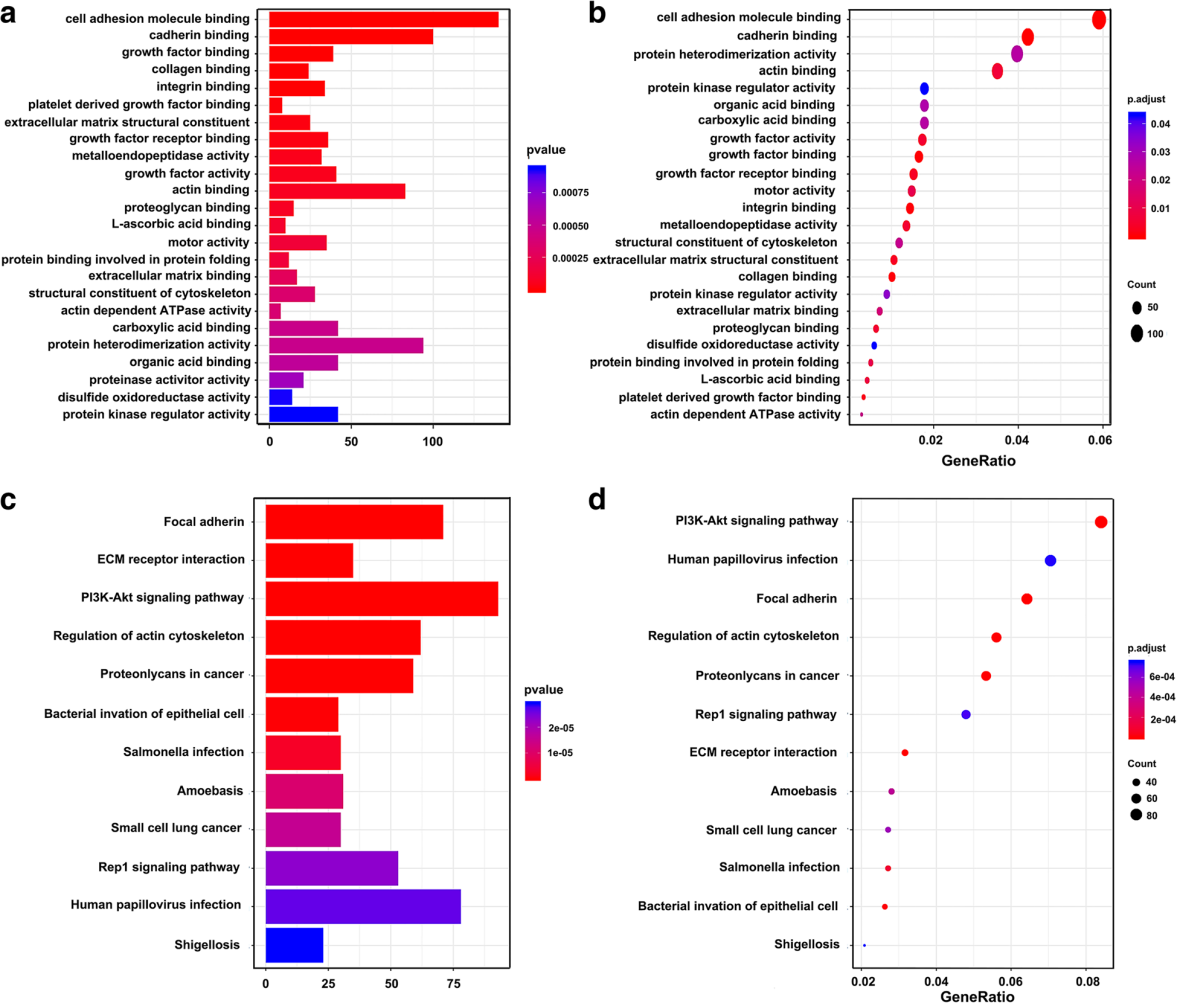
GO and KEGG pathway enrichment analysis for MYOSLID-related mRNAs based on TCGA data. **a** and **b** Plot of the enriched GO terms Go enrichment analysis for MYOSLID-related mRNAs. Y-axis represents the enriched GO terms; X-axis (**a**) represents the amount of the MYOSLID-related mRNAs enriched in GO terms; X-axis (**b**) represents the ratio of the MYOSLID-related mRNAs enriched inGO terms. **b**, **c** and **d** Plot of the KEGG pathways KEGG pathway enrichment analysis for MYOSLID-related mRNAs. Y-axis represents pathways; X-axis (**c**) represents the amount of the MYOSLID-related mRNAs enriched in KEGG pathways; X-axis (**d**) represents the ratio of the MYOSLID-related mRNAs enriched in KEGG pathways. The color and size of each bubble represent enrichment significance and the number of MYOSLID-related mRNAs enriched in a GO term or pathway, respectively. *p* < 0.05 was usedas the threshold to select GO and KEGG terms. GO, Gene Ontology; KEGG, Kyoto Encyclopedia of Genes and Genomes

MYOSLID-related mRNAs (|Pearson correlation coefficient| > 0.3) were selected for GO and KEGG pathway analysis to further explore their biological function. A total of 24 GO terms and 33 pathways (*p* < 0.05) were identified, as shown in Fig. 2, Additional file 1: Table S4 and S5. The most significantly enriched GO terms for MYOSLID were cell adhesion molecule binding, cadherin binding and protein heterodimerization, as shown in Fig. 2a. Similarly, the significant pathways for MYOSLID-related mRNAs were mainly enriched in the PI3K/AKT signaling pathway, human papillomavirus infection, focal adhesion and regulation of the actin cytoskeleton, as shown in Fig. 2b.

### Expression of MYOSLID in fresh OSCC tissue specimens and OSCC cell lines

**Fig. 3:**
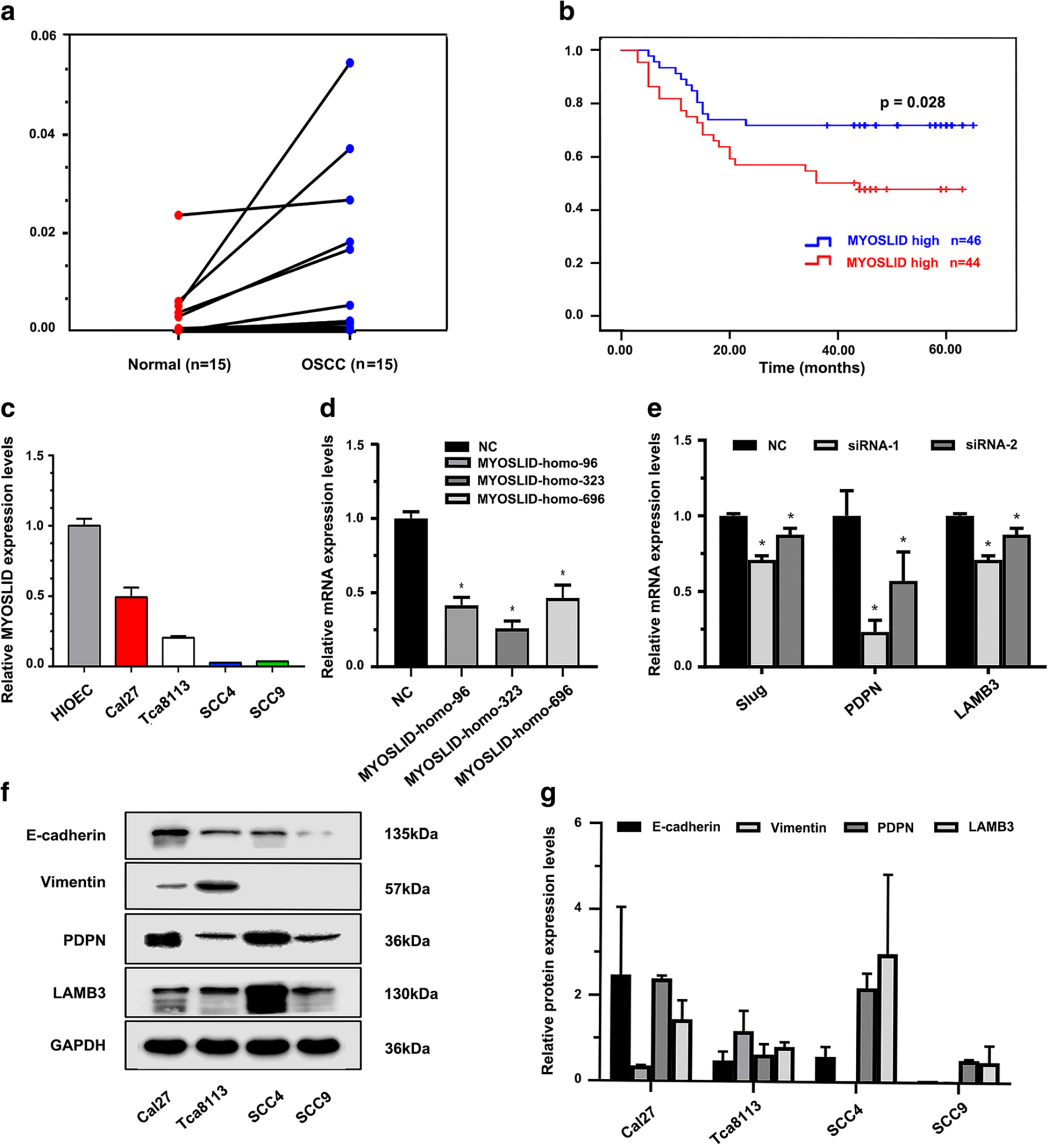
Validation of MYOSLID expression in OSCC specimens and cell lines. **a** RT-qPCR validation of MYOSLID expression in 15 paired OSCC tissues and adjacent normal mucosa; a simple t test was used to test the significance of the differences between the 2 groups, *p* < 0.05. **b** Survival analysis of MYOSLID expression in an HNSCC microarray assay, *p* < 0.05. **c** MYOSLID expression in 4 OSCC cell lines. **d** siRNA sequences targeting the MYOSLID sequence. **e** Effect of silencing MYOSLID on the mRNA expression levels of p-EMT-related markers. **f**-**g** Classical EMT marker and p-EMT marker protein levels in 4 OSCC cell lines. siRNA-1 refers to MYOSLID-homo-96, and siRNA-2 refers to MYOSLID-homo-323

We detected MYOSLID expression in 15 paired fresh OSCC cancer tissues and adjacent normal tissues by RT-qPCR. The results showed that MYOSLID expression in the cancer group (0.01127 ± 0.01655) was higher than that in the adjacent cancer normal tissues (0.00299 ± 0.00608) (*p* = 0.037) (Fig. 3a). Meanwhile, we detected the MYOSLID expression levels in a normal oral epithelium cell line, HIOEC, and 4 OSCC cell lines, Cal27, Tca8113, SCC4, and SCC9. The results showed that the Cal27 cell line had a higher MYOSLID expression level (0.49254 ± 0.07004) compared with that in Tca8113 (0.20549 ± 0.01073), SCC4 (0.02700 ± 0.00008), and SCC9 (0.03700 ± 0.00009) (*p* < 0.05). Unexpectedly, we found that the human normal oral epithelium cell line HIOEC had the highest MYOSLID expression levels of all of the cell lines that were examined (Fig. 3c).

### Evaluation of the clinical significance of MYOSLID expression in a human HNSCC tissue microarray

The results of the Kaplan-Meier survival analysis showed that patients with higher MYOSLID expression had a poor prognosis (log rank [Mantel-Cox] = 4.858, *p* = 0.028), as show in Fig. 3b.

**Fig. 4:**
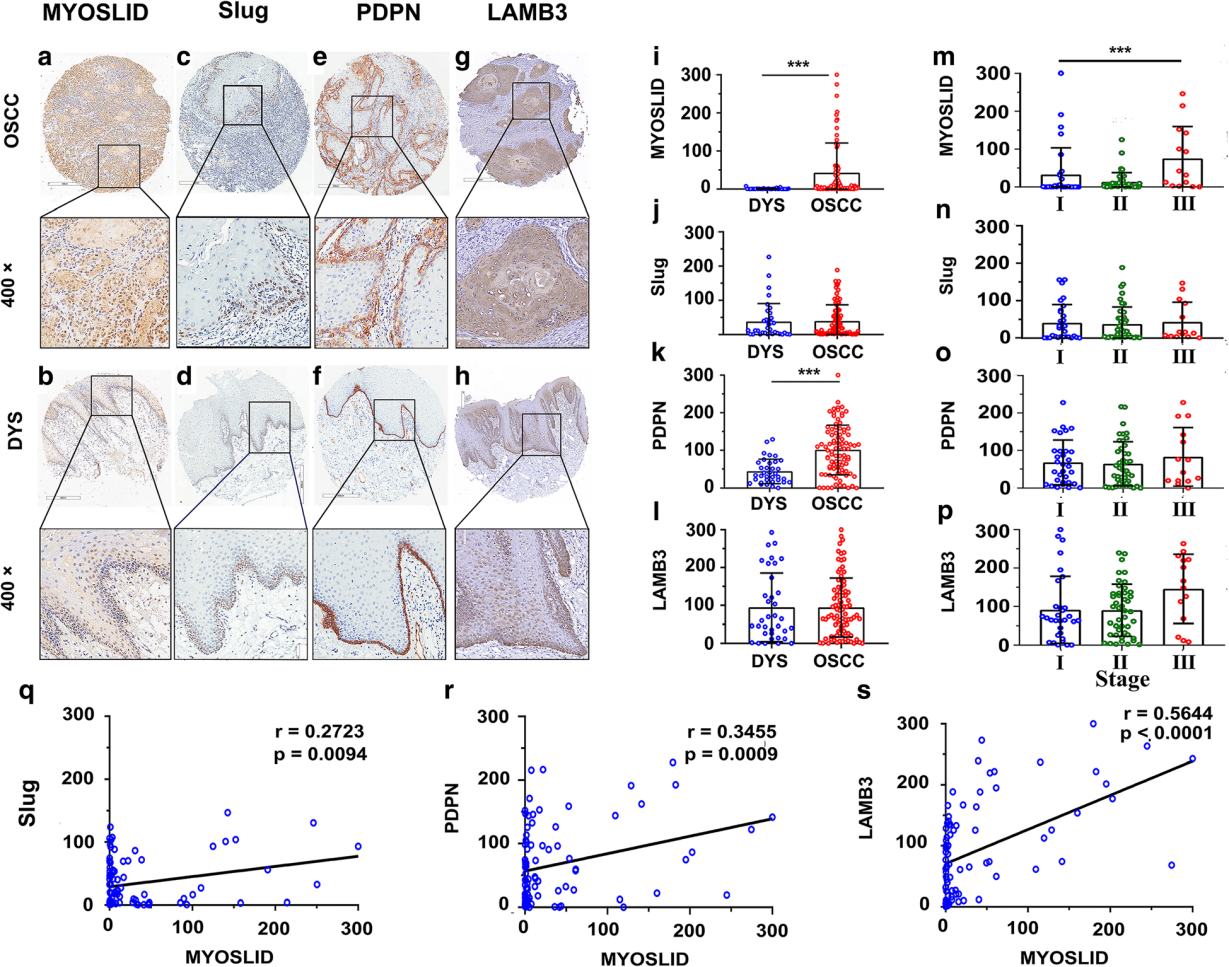
MYOSLID and p-EMT regulator expression in human HNSCC tissue microarray and its clinical significance. **a-h** Representative images of MYOSLID ISH and Slug, PDPN, LAMB3 IHC in OSCC and DYS tissues (upper: magnification × 100, lower: magnification × 400). **i-l** Histoscore of MYOSLID, Slug, PDPN and LAMB3 in OSCC tissues and the DYS group. A t test was conducted to identify significant differences. *P* < 0.05 was considered significant. MYOSLID (t = 3.271, *p* < 0.0014) and PDPN (t = 4.866, *p* < 0.0001) expression in the OSCC group was significantly higher than that in the DYS group. **m-p** One-way ANOVA was conducted to determine the differences in MYOSLID, Slug, PDPN and LAMB3 expression in the various stages. The results show that MYOSLID expression was related to advanced clinical stage (F = 6.701, *p* = 0.002). **q-s** A Pearson correlation coefficient was calculated to determine the correlation among MYOSLID, Slug, PDPN and LAMB3. *p* < 0.05 was considered significant

The results of chi-square χ^2^ showed that there were no significant relationship between MYOSLID expression and the clinical pathological characteristics (Table 3). However, the results of the ISH histoscore of MYOSLID expression showed that MYOSLID (t = 3.271, *p* = 0.0014) and PDPN expression (t = 4.866, *p* < 0.0001) in the OSCC group was significantly higher than that in the DYS group. Advanced OSCC patients had higher MYOSLID expression levels than those in early stage patients (F = 6.701, *p* = 0.0020) (Fig. 4a). Notably, subcellular localization analysis showed that MYOSID was expressed in both the cytoplasm and nucleus (Fig. 4a).

**Table 3:** MYOSLID expression and clinicopathological Characteristics of patients with HNSCC(*n* = 90)

Characteristics	MYOSLID		χ2	*p* value
	Low expression (*n* = 46)	High expression (*n* = 44)		
Age(y)				
< 60	23	26	0.749	0.387
≥ 60	23	18		
Gender				
Male	41	34	2.277	0.131
Female	5	10		
Smoke				
Yes	27	20	1.580	0.209
No	19	24		
Drink				
Yes	23	21	0.046	0.829
No	23	23		
Stage				
Stage I/II	39	38	0.045	0.831
Stage III/IV	7	6		
T				
T_1_ + T_2_	27	28	2.985	0.394
T_3_ + T_4_	19	16		
N				
Yes	30	25	0.472	0.492
No	16	18		

Note: HNSCC patient were divided into MYOSLID low and MYOSLID high group according to the cut off value (median = 2.453)

*p* < 0.05 was considered statistic significant. Differences among variables were evaluated by χ2 or Fisher’s exact χ2 -test

Univariate analysis indicated that MYOSLID expression (HR = 2.096, 95%CI [1.061–4.140], *p* = 0.033) and N condition (HR = 2.981, 95%CI [1.513–5.875], *p* = 0.002) were related to OS in OSCC patients. Multivariate Cox analysis indicated that MYOSLID expression (HR = 2.223, 95%CI [1.079–4.580], *p* = 0.030) and N condition (HR = 2.353, 95%CI [1.136–4.877], *p* = 0.021) were both independent prognostic risk factors related to OS, as show in Table 4.

**Table 4:** Univariate and multivariate Cox regression analysis for OS in HNSCC patients (*n* = 90)

Variable	Univariate analysis			Multivariate analysis		
	*p*	HR	95% CI	*p*	HR	95% CI
MYOSLID	0.033	2.096	1.061–4.140	0.030	2.223	1.079–4.580
Age	0.128	1.667	0.863–3.219	0.056	2.029	0.982–4.195
Gender	0.130	1.793	0.843–3.816	0.799	1.146	0.403–3.252
Smoke	0.985	1.006	0.523–1.936	0.705	1.189	0.486–2.905
Drink	0.193	0.641	0.328–1.253	0.228	0.608	0.271–1.366
Stage	0.265	1.601	0.700–3.659	0.333	1.575	0.628–3.950
T	0.059	1.426	0.987–2.060	0.101	1.469	0.928–2.324
N	0.002	2.981	1.513–5.875	0.021	2.353	1.136–4.877

Notes: *N* Regional Lymph Nodes, *T* Primary Tumor, *OS* overall survival

### The expression levels of MYOSLID were correlated with slug, PDPN and LAMB3 in a human HNSCC tissue microarray

Pearson correlation analysis revealed that the expression level of MYOSLID was correlated with Slug (*r* = 0.2723, *p* = 0.0094), PDPN (*r* = 0.3455, *p* = 0.0009) and LAMB3 (*r* = 0.5644, *p* = 0.0001) expression, as shown in Fig. 4c.

### Knockdown MYOSLID inhibited OSCC cell migration and invasion

The expression level of MYOSLID was the highest in Cal27 cell line, and Cal27 cell line had a co-expression of p-EMT makers (PDPN, LAMB3) and classical EMT markers (E-cadherin, Vimentin) (Fig. 3c, f, g). This indicates that the state of the Cal27 cell line is the closest to a p-EMT. The two siRNA sequences MYOSLID-homo-96 and MYOSLID-homo-232 were selected to knockdown MYOSLID in Cal27 cell lines, as they were validated to have a higher knockout efficiency compared with that of MYOSLID-homo-696 (Fig. 3d). Wound healing assays showed that the knockdown of MYOSLID expression significantly reduced the 48 h healing rate (*p* < 0.05, Fig. 4a). Moreover, the transwell assay showed that the number of cells passed through the filter that was coated with Matrigel of the siRNA groups was much lower than that of the negative control group (*p* < 0.05, Fig. 4b).

### Knockdown MYOSLID inhibited Slug, PDPN and LAMB3 expression

**Fig. 5:**
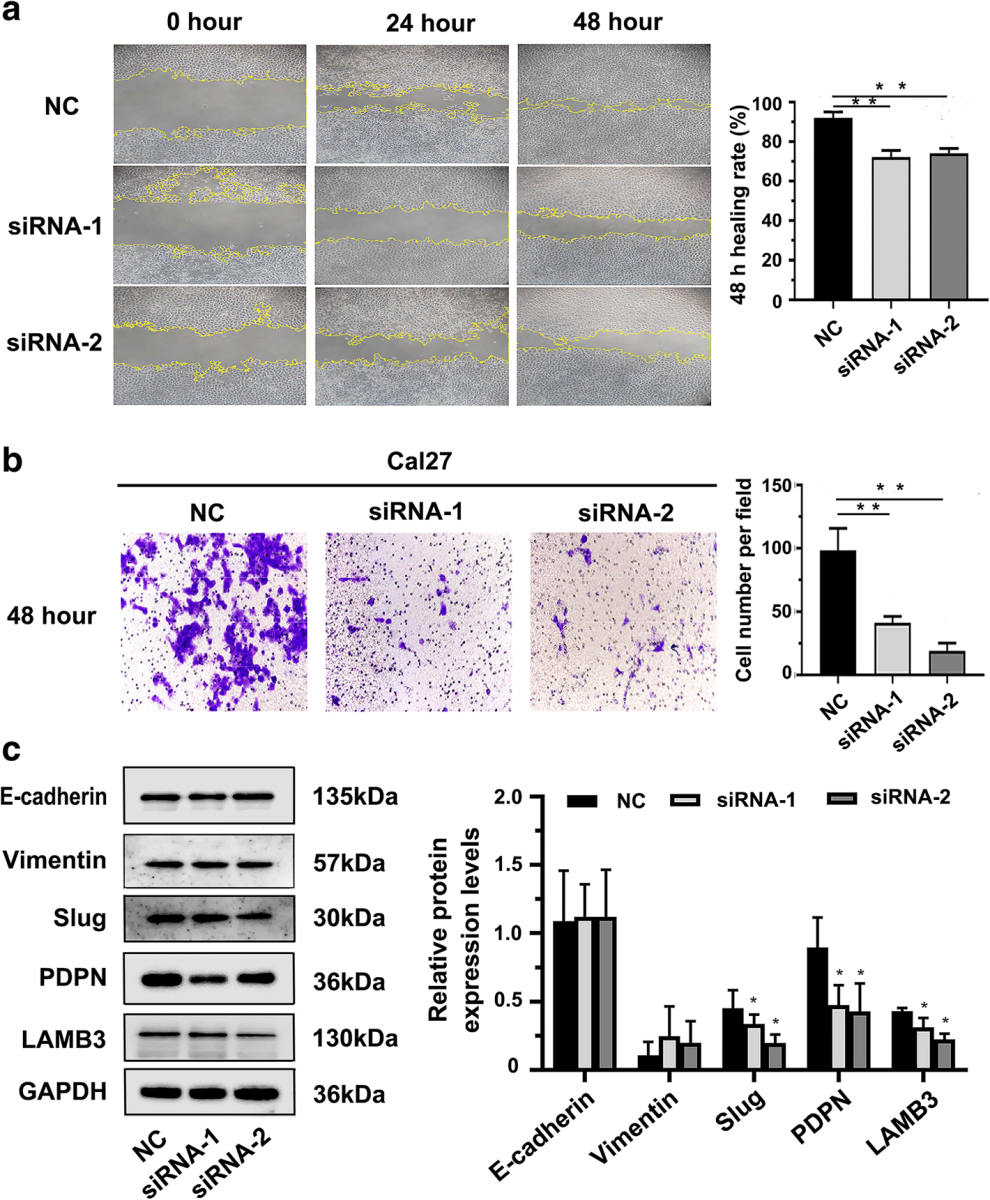
Effect of MYOSLID on the invasion and metastasis of OSCC cell lines. **a** Effect of MYOSLID on the cell migration of Cal27, as determined by wound healing assays. Images of the wound healing assays of the 2 siRNA groups and the negative control group were captured at 0 h, 24 h and 48 h (magnification, × 10). Bar graph indicates the mean healing rate of the 3 experimental repetitions of each group. **b** Effect of MYOSLID on cell invasion was determined by transwell assay. Representative images of the siRNA group and negative control group were captured after 48 h (magnification, × 20). Bar graph indicates the mean number of cells that passed through the filter coated with Matrigel. Three repeated experiments were performed for each group. **c** Results of Western blot showed that knockdown MYOSLID in Cal27 cell line caused a significant downregulation of p-EMT related markers Slug, PDPN and LAMB3 protein levels, but protein levels of Classical EMT related markers E-cadherin and Vimentin remain unchanged. “*” indicates that there was statistical significance indicated by a two-sample t test. *p* < 0.05 was considered statistically significant. The results are presented as the mean ± SEM (*n* = 3). siRNA-1 refers to MYOSLID-homo-96, and siRNA-2 refers to MYOSLID-homo-323

To further verify the molecular mechanicals of aberrant MYOSLID expression affects the ability of invasion and metastasis of OSCC cells was by regulating the p-EMT program. Expression levels of p-EMT markers Slug, PDPN, LAMB3 and EMT markers E-cadherin, Vimentin were detected after knockdown MYOSLID in OSCC cell line. The results showed that knockdown MYOSLID expression significantly inhibited Slug, PDPN and LAMB3 mRNA and protein expression levels compared to those in the controls (*p* < 0.05, Fig. 3e, Fig. 5c). Interestingly, the expression levels of the classical EMT biomarkers E-cadherin and Vimentin remained unchanged after silencing MYOSLID (Fig. 5c).

## Discussion

On the one hand, bioinformatics analysis has been widely used to uncover the genetic changes in the high-throughput data from tumors. On the other hand, the p-EMT process was observed in a highly invasive HNSCC with a single-cell sequencing technique [8]. The Slug gene was identified as a key gene in the regulation of the p-EMT process [9]. Here, we identified MYOSLID as a lncRNA that was highly correlated with the expression of Slug and with a prognostic value in HNSCC using bioinformatics analysis. GO and KEGG function enrichment analysis of MYOSLID-related mRNAs suggested that MYOSLID expression in HNSCC was associated with many biologic processes, such as cell adhesion molecule binding, cadherin binding, growth factor binding, collagen binding. Most of the enriched pathways or biologic processes have been reported to be involved in regulating cancer metastasis [24–26].

MYOSLID is a long noncoding RNA located in a lncRNA-rich genome region on chromosome 2. Studies on MYOSLID in cancer have never been reported. MYOSLID was first reported as a long noncoding RNA that is related to the differentiation program of vascular smooth muscle cells (VSMCs), and the knockdown of MYOSLID with siRNA in human coronary artery SMCs (HCASMCs) disrupted the formation of the actin cytoskeleton [27]. Although the function of MYOSLID in epithelial cell types has not been explored, it is reasonable to speculate that MYOSLID expression in epithelial cells may also be related to the cancer cell differentiation program.

This study aimed to understand the function and clinical significance of MYOSLID expression in HNSCC. First, we validated the upregulation of MYOSLID in HNSCC tissues compared with adjacent normal tissue by RT-qPCR. Then, MYOSLID expression was detected in a human HNSCC microarray by ISH. The results showed that MYOSLID expression was associated with poor prognosis in HNSCC. Higher MYOSLID expression was related to advanced TNM stage and lymph node metastasis. This was consistent with the results of bioinformatics analysis of data from TCGA. Therefore, MYOSLID may serves as an oncogene in HNSCC. The function of lncRNA can be predicted by its localization in cells. Subcellular localization analysis showed that MYOSLID was expressed at both cytoplasm and nuclei. However, Zhao et al. reported that most MYOSLID expression was localized in the cytoplasm of VSMCs [27]. This result indicates that MYOSLID may have different functions in cancer cells compared with those in VSMCs.

A list of predicted MYOSLID target genes showed that the Slug gene was at one of the mRNAs that was most strongly related to MYOSLID. Slug is one of the major classical transcription factors (TFs) that drive EMT. E-cadherin, N-cadherin and Vimentin are downstream genes of Slug [28]. However, we did not find E-cadherin, N-cadherin and Vimentin among the list of predicted MYOSLID target genes. Surprisingly, we found that MYOSLID expression was closely correlated with PDPN and LAMB3. Then, we performed IHC for Slug, PDPN and LAMB3 in a human HNSCC microarray. We observed that the PDPN and LAMB3 proteins were specifically expressed in OSCC cells at the tumor periphery. The results of the correlation analysis also indicated that MYOSLID expression was closely correlated with Slug, PDPN and LAMB3. PDPN and LAMB3 are specific p-EMT markers [8]. Leroy P noted that Slug loses the ability to regulate E-cadherin expression during the p-EMT phase [9]. All of this suggested to us that MYOSLID expression in HNSCC might be related to the function of Slug in controlling the p-EMT program instead of the EMT program.

Thus, we further validated and confirmed the functions of MYOSLID in controlling the p-EMT program. We knocked down MYOSLID with siRNA in the Cal27 cell line, as it expressed both the p-EMT markers PDPN and LAMB3 and the epithelial adhesion junction protein E-cadherin. The knockdown of MYOSLID caused a reduction in the expression of Slug, PDPN and LAMB3, but the expression levels of E-cadherin and Vimentin remain unchanged, compared to those in the controls. This further supports our previous hypothesis that MYOSLID expression is mainly responsible for maintaining the functions of Slug in regulating the p-EMT program. Wang et al. demonstrate that the knockdown of Slug in the OSCC cell lines UM1 and SCC9 caused an upregulation of E-cadherin and a downregulation of Vimentin. However, Slug knockdown in the SCC15 cell line did not affect E-cadherin and Vimentin expression [29]. This may be explained by the fact that these cell lines are in different EMT phases and that Slug performs different functions. Similarly, our speculation was also supported by Wicki A et al.’s work, in which they overexpressed PDPN in a double transgenic Rip1Podo:Rip1Tag2 mouse model of carcinogenesis to transform a benign adenoma into an invasive carcinoma, in which the complete loss of E-cadherin and the upregulation of N-cadherin were not detected during progression [7]. At present, there are still many unsolved problems regarding the detailed molecular mechanisms of p-EMT. Contrary to our results, some researchers observed different phenomena after deleting PDPN or LAMB3. For example, Asai et al. reported that the downregulation of PDPN in normal human epidermal keratinocytes (NHEKs) caused the upregulation of E-cadherin [30]. Liu et al. reported that the downregulation of LAMB3 in the HNSCC cell lines SNU1041 and SNU1076 increased E-cadherin expression but reduced Vimentin and Slug expression [31]. This may be explained by p-EMT being a metastable and reversable state between the epithelial and mesenchymal states. Inhibiting key genes that control p-EMT will transition the cells back into a non-p-EMT state that is close to the epithelial state.

There are some limitations to this study. We only performed a preliminary study on the functions of MYOSLID in an OSCC cell line that had relatively aggressive features. The specific cells in the p-EMT phase should be sorted for unbiased analysis. The direct interaction between MYOSLID and Slug also needs to be further investigated.

## Conclusions

In summary, we revealed that the upregulation of MYOSLID in HNSCC was associated with poor prognosis. Our results also demonstrated that inhibiting MYOSLID expression significantly inhibited cancer cell invasion and metastasis. Most importantly, the aberrant expression of MYOSLID has no influence on classical EMT markers but does influence the expression levels of p-EMT-related markers. This indicates that MYOSLID is a valuable biomarker of aggressive HNSCC. The detection of MYOSLID expression may be meaningful for guiding the selection of clinical treatment options for HNSCC. MYOSLID may be a promising target for controlling cancer metastasis.

## Additional files

Additional file 1: **Table S1.** Details of the samples used in the RT-qPCR experiment. **Table S2.** List of primers used for RT-qPCR and sequences of designed MYOSLID small interfere RNA. **Table S3.** Clinical pathological characteristics of HNSCC patients from the TCGA database (*n* = 499). **Table S4.** The GO analysis of the predicted target genes of MYOSLID. **Table S5.** KEGG pathway analysis of the predicted target genes of MYOSLID. (DOCX 28 kb)

Additional file 2: R script that use the “edgeR” package for differential expression analysis. (TXT 2 kb)

Additional file 3: R script that use the “limma” package for differential expression analysis. (TXT 1 kb)

## Data Availability

All the data and materials supporting the conclusions were included in the main paper.

## Abbreviations

95%CI: 95% confidence interval
DEGs: Differentially expressed mRNAs
DE-lncRNAs: Differentially expressed lncRNAs
EMT: Epithelial mesenchymal transition
GEO: Gene Expression Omnibus
GO: Gene Ontology
HNSCC: Head and neck squamous cell carcinoma
HR: Hazard ratio
ISH: In Site Hybridization
KEGG: Kyoto Encyclopedia of Genes and Genomes
LAMB3: Laminin subunit beta 3
MYOSLID: MYOcardin-induced Smooth muscle Long noncoding RNA, Inducer of Differentiation
OS: Overall Survival
OSCC: Oral squamous cell carcinoma
PDPN: Podoplanins
p-EMT: Partial epithelial mesenchymal transition
RT-qPCR: Real time quantitative polymerase chain reaction
TCGA: The Cancer Genome Atlas
TFs: Transcription factors
VSMC: Vascular smooth muscle cell
