# Supplementary material for: Long noncoding RNA MYOSLID promotes invasion and metastasis by modulating the partial epithelial-mesenchymal transition program in head and neck squamous cell carcinoma
Source: J Exp Clin Cancer Res. 2019 Jun 25;38:278. doi: 10.1186/s13046-019-1254-4 (PMC6593600; doi:10.1186/s13046-019-1254-4)
Supplement: Supplementary file 1 — Table S1. Details of the samples used in the RT-qPCR experiment. Table S2. List of primers used for RT-qPCR and sequences of designed MYOSLID small interfere RNA. Table S3. Clinical pathological characteristics of HNSCC patients from the TCGA database (n = 499). Table S4. The GO analysis of the predicted target genes of MYOSLID. Table S5. KEGG pathway analysis of the predicted target genes of MYOSLID. (DOCX 28 kb) [file 13046_2019_1254_MOESM1_ESM.docx]

Table S1. Clinical pathological characteristics of 15 HNSCC patients.

| Characteristics | Number of cases (%) |
| --- | --- |
| **Age(y)** |  |
| <60 | 9(60%) |
| ≥60 | 6(40%) |
| **Gender** |  |
| Male | 12(80.0%) |
| Female | 3(20%) |
| **Smoke** |  |
| Yes | 8（53.3%） |
| No | 7（46.7%） |
| **Drink** |  |
| Yes | 11(73.3%) |
| No | 4(26.7%) |
| **Stage** |  |
| Stage I/II | 13(86.7%) |
| Stage III/IV | 2(13.3%) |
| **T** |  |
| T1/2 | 13(86.7%) |
| T3/4 | 2(13.3%) |
| **N** |  |
| Yes | 5 (33.3%) |
| No | 10(66.7% |
| Note: N, Regional Lymph Nodes; T, Primary Tumor; M, distant metastasis.HNSCC patient were divided into MYOSLID low and MYOSLID high group according to the cut off value (median=2.453).  Abbreviations: HNSCC, head and neck squamous cell carcinoma. | |

Table S2. List of primers used for RT-qPCR and sequences of designed MYOSLID small interfere RNA.

| **Genes** | **Sequences (**5’-3’**)** |
| --- | --- |
| MYOSLID | Forward primer:5’-AAGAGGGAGTGGGAGTTAGGC-3’ |
|  | Reverse primer:5’-CACTGTGGTGGGATCTGCAAG-3’ |
| Slug | Forward primer:5’-ACTCACTCGCCCCAAAGATG-3’ |
|  | Reverse primer:5’-CAACGCCTCCAAAAAGCCAA-3’ |
| LAMB3 | Forward primer:5’- CAGCATGGCCGTGACAGAA-3’ |
|  | Reverse primer:5’- CAGGATGTTCGGTGCCAGTC-3’ |
| PDPN | Forward primer:5’- CCTCGATGCGAATGCCTGTTA-3’ |
|  | Reverse primer:5’- GGTGCCGAAGATGATGTGGT-3’ |
| GAPDH | Forward primer:5’- TGATGACCCTTTTGGCTCCC-3’ |
|  | Reverse primer:5’- GAAGCTTGTCATCAATGGAAAT-3’ |
| MYOSLID-homo-96 | 5’-GCUCAAGUCAAACGCAUUUTTAAAUGCGUUUGA  CUUGAGCTT-3’ |
| MYOSLID-homo-323 | 5’-GGACAUGGCUGAGCAUGUUTTAACAUGCUCAGC  CAUGUCCTT-3’ |
| MYOSLID-homo-696 | 5’-GCUGCAUCGCAGUUUACUUTTAAGUAAAACUGC  GAUGCAGCTT-3’ |

Table S3. Clinical pathological characteristics of HNSCC patients from the TCGA database (n=499).

| Characteristics | Number of cases (%) |
| --- | --- |
| **Age(y)** |  |
| <60 | 221(44.3%) |
| ≥60 | 278(55.7%) |
| **Gender** |  |
| Male | 366(73.3%) |
| Female | 133(26.7%) |
| **Race** |  |
| White | 427（85.6%） |
| Black | 47（9.4%） |
| Asian | 10（2.0%） |
| unknow | 15(3%) |
| **Grade** |  |
| G1/2 | 360(72.1%) |
| G3/4 | 120(24.0%) |
| Gx | 3(0.6%) |
| **Stage** |  |
| Stage I/II | 96(19.2%) |
| Stage III/IV | 335(67.1%) |
| unknow | 68(13.6%) |
| **T** |  |
| T1/2 | 215(43.1%) |
| T3/4 | 262(52.5%) |
| unknow | 22(4.4%) |
| **N** |  |
| Yes | 239(47.9%) |
| No | 236(47.3%) |
| unknow | 14(4.8%) |
| **M** |  |
| M0 | 185 (37.1%) |
| M1 | 1 (0.2%) |
| Mx | 61 (12.2%) |
| unknow | 252 (50.5%) |
| Note: N, Regional Lymph Nodes; T, Primary Tumor; M, distant metastasis.  Abbreviations: TCGA, the Cancer Genome Atlas; HNSCC, head and neck squamous cell carcinoma. | |

Table S4. The GO analysis of the predicted target genes of MYOSLID.

| ID | Description | p.adjust | Count |
| --- | --- | --- | --- |
| GO:0050839 | cell adhesion molecule binding | 5.81E-18 | 140 |
| GO:0045296 | cadherin binding | 2.33E-15 | 100 |
| GO:0019838 | growth factor binding | 0.000353 | 39 |
| GO:0005518 | collagen binding | 0.000595 | 24 |
| GO:0005178 | integrin binding | 0.000616 | 34 |
| GO:0048407 | platelet-derived growth factor binding | 0.002463 | 8 |
| GO:0005201 | extracellular matrix structural constituent | 0.003601 | 25 |
| GO:0070851 | growth factor receptor binding | 0.003601 | 36 |
| GO:0004222 | metalloendopeptidase activity | 0.005579 | 32 |
| GO:0008083 | growth factor activity | 0.005579 | 41 |
| GO:0003779 | actin binding | 0.006511 | 83 |
| GO:0043394 | proteoglycan binding | 0.006511 | 15 |
| GO:0031418 | L-ascorbic acid binding | 0.009337 | 10 |
| GO:0003774 | motor activity | 0.01088 | 35 |
| GO:0044183 | protein binding involved in protein folding | 0.01088 | 12 |
| GO:0050840 | extracellular matrix binding | 0.018926 | 17 |
| GO:0005200 | structural constituent of cytoskeleton | 0.022649 | 28 |
| GO:0030898 | actin-dependent ATPase activity | 0.022649 | 7 |
| GO:0031406 | carboxylic acid binding | 0.02637 | 42 |
| GO:0046982 | protein heterodimerization activity | 0.02637 | 94 |
| GO:0043177 | organic acid binding | 0.02782 | 42 |
| GO:0030295 | protein kinase activator activity | 0.033743 | 21 |
| GO:0015036 | disulfide oxidoreductase activity | 0.043092 | 14 |
| GO:0019887 | protein kinase regulator activity | 0.043092 | 42 |

Abbreviations: GO, Gene Ontology.

Table S5. KEGG pathway analysis of the predicted target genes of MYOSLID.

| ID | Description | p.adjust | Count |
| --- | --- | --- | --- |
| hsa04510 | Focal adhesion | 3.28E-11 | 71 |
| hsa04512 | ECM-receptor interaction | 1.34E-07 | 35 |
| hsa04151 | PI3K-Akt signaling pathway | 6.30E-07 | 93 |
| hsa04810 | Regulation of actin cytoskeleton | 3.37E-06 | 62 |
| hsa05205 | Proteoglycans in cancer | 4.14E-06 | 59 |
| hsa05100 | Bacterial invasion of epithelial cells | 1.19E-05 | 29 |
| hsa05132 | Salmonella infection | 0.000115 | 30 |
| hsa05146 | Amoebiasis | 0.000429 | 31 |
| hsa05222 | Small cell lung cancer | 0.000539 | 30 |
| hsa04015 | Rap1 signaling pathway | 0.00072 | 53 |
| hsa05165 | Human papillomavirus infection | 0.000747 | 78 |
| hsa05131 | Shigellosis | 0.000754 | 23 |
| hsa03050 | Proteasome | 0.000779 | 18 |
| hsa04360 | Axon guidance | 0.000997 | 46 |
| hsa05418 | Fluid shear stress and atherosclerosis | 0.00171 | 38 |
| hsa05130 | Pathogenic Escherichia coli infection | 0.003941 | 19 |
| hsa04926 | Relaxin signaling pathway | 0.003941 | 35 |
| hsa04390 | Hippo signaling pathway | 0.006318 | 39 |
| hsa04012 | ErbB signaling pathway | 0.006318 | 25 |
| hsa04114 | Oocyte meiosis | 0.006318 | 33 |
| hsa04010 | MAPK signaling pathway | 0.011147 | 64 |
| hsa05230 | Central carbon metabolism in cancer | 0.011147 | 20 |
| hsa04933 | AGE-RAGE signaling pathway in diabetic complications | 0.011752 | 27 |
| hsa04971 | Gastric acid secretion | 0.011752 | 22 |
| hsa04974 | Protein digestion and absorption | 0.0126 | 25 |
| hsa00532 | Glycosaminoglycan biosynthesis - chondroitin sulfate / dermatan sulfate | 0.014123 | 9 |
| hsa05219 | Bladder cancer | 0.017992 | 14 |
| hsa04914 | Progesterone-mediated oocyte maturation | 0.021926 | 26 |
| hsa04915 | Estrogen signaling pathway | 0.027412 | 33 |
| hsa01521 | EGFR tyrosine kinase inhibitor resistance | 0.04527 | 21 |
| hsa05110 | Vibrio cholerae infection | 0.04527 | 15 |
| hsa05163 | Human cytomegalovirus infection | 0.045974 | 48 |

Abbreviations: Kyoto Encyclopedia of Genes and Genomes; ECM, extracellular matrix.
